# Silencing Status Epilepticus-Induced *BDNF* Expression with Herpes Simplex Virus Type-1 Based Amplicon Vectors

**DOI:** 10.1371/journal.pone.0150995

**Published:** 2016-03-08

**Authors:** Chiara Falcicchia, Pascal Trempat, Anna Binaschi, Coline Perrier-Biollay, Paolo Roncon, Marie Soukupova, Hervé Berthommé, Michele Simonato

**Affiliations:** 1 Department of Medical Science, Section of Pharmacology, Neuroscience Center, University of Ferrara and National Institute of Neuroscience, Ferrara, Italy; 2 Bioviron, Université Claude Bernard Lyon 1, Villeurbanne, France; 3 Laboratory of Technologies for Advanced Therapy (LTTA), Technopole of Ferrara, Ferrara, Italy; University of Modena and Reggio Emilia, ITALY

## Abstract

Brain-derived neurotrophic factor (BDNF) has been found to produce pro- but also anti-epileptic effects. Thus, its validity as a therapeutic target must be verified using advanced tools designed to block or to enhance its signal. The aim of this study was to develop tools to silence the BDNF signal. We generated Herpes simplex virus type 1 (HSV-1) derived amplicon vectors, i.e. viral particles containing a genome of 152 kb constituted of concatameric repetitions of an expression cassette, enabling the expression of the gene of interest in multiple copies. HSV-1 based amplicon vectors are non-pathogenic and have been successfully employed in the past for gene delivery into the brain of living animals. Therefore, amplicon vectors should represent a logical choice for expressing a silencing cassette, which, in multiple copies, is expected to lead to an efficient knock-down of the target gene expression. Here, we employed two amplicon-based BDNF silencing strategies. The first, antisense, has been chosen to target and degrade the cytoplasmic mRNA pool of BDNF, whereas the second, based on the convergent transcription technology, has been chosen to repress transcription at the *BDNF* gene. Both these amplicon vectors proved to be effective in down-regulating *BDNF* expression *in vitro*, in BDNF-expressing mesoangioblast cells. However, only the antisense strategy was effective *in vivo*, after inoculation in the hippocampus in a model of status epilepticus in which BDNF mRNA levels are strongly increased. Interestingly, the knocking down of BDNF levels induced with BDNF-antisense was sufficient to produce significant behavioral effects, in spite of the fact that it was produced only in a part of a single hippocampus. In conclusion, this study demonstrates a reliable effect of amplicon vectors in knocking down gene expression *in vitro* and *in vivo*. Therefore, this approach may find broad applications in neurobiological studies.

## Introduction

The neurotrophin brain-derived neurotrophic factor (BDNF) is widely expressed in the brain, where it exerts a key role in neuronal survival, differentiation, and plasticity [1,2]. BDNF is therefore viewed as a promising therapeutic target for central nervous system disorders, for example in the transformation of a normal brain in epileptic, a phenomenon in which cell death, neurogenesis and morphological and functional changes are thought to be involved [3,4]. However, an important issue that has limited development of therapies that target the BDNF system has been the difficulty in delivering it to a specific and restricted brain region and thereby locally modulating its levels. Here, we present novel tools to pursue this aim: amplicon vectors derived from Herpes simplex virus type 1 (HSV-1) capable to down-regulate *BDNF* expression *in vitro* and *in vivo*.

Amplicon vectors are HSV-1 particles identical to wild type HSV-1 from the structural and host-range points of view, but which carry a concatemeric form of a DNA plasmid, named amplicon plasmid, instead of the 152 Kb viral genome. HSV-1 amplicon vectors hold considerable promise as gene-transfer vehicles because of their very high capacity to host foreign DNA with high number of repeats of the transgene [5], a feature that, in principle, makes them particularly suited to efficiently knock-down a target gene.

Two silencing strategies have been pursued in this study. The first, called “antisense”, has been chosen to target and degrade the cytoplasmic messenger RNA (mRNA) pool of BDNF via an RNA interference (RNAi) mechanism [6]. In RNAi, antisense RNA molecules inhibit gene expression by causing the destruction of complementary mRNA molecules in a sequence-specific manner [7]. A natural antisense BDNF mRNA has been recently identified in the mouse brain [8].

The second strategy, based on the “convergent transcription technology” [9], represses the *BDNF* gene through chromatin remodeling. The convergent transcription approach is based on co-expression of the sense and antisense RNA strands from independent expression cassettes or a divergent cassette in which a full-length complementary DNA (cDNA) sequence is positioned between two identical promoters [10,11], such that independent transcription from each promoter produces a pool of sense and antisense RNAs capable of forming long dsRNAs and undergoing processing to the effector siRNAs [9]. The use of convergent transcription from opposing promoters to induce gene silencing has been reported in trypanosomes and Drosophila [12,13], as well as in yeast and mammalian cells [14]. It has been predicted that the expression of up to 8% of human genes may be influenced by antisense RNA or antisense transcription [15,16], suggesting that convergent transcription does occur with high frequency in the human genome [9].

In the present study, the silencing effect of amplicon vectors has been assessed by examining their efficiency in down-regulating BDNF levels *in vitro*, in BDNF-expressing mesoangioblast cells, and *in vivo*, using a rat model of status epilepticus in which BDNF mRNA levels are strongly increased in the hippocampus.

## Materials and Methods

### Amplicon vectors

#### Amplicon plasmids

**Fig 1:**
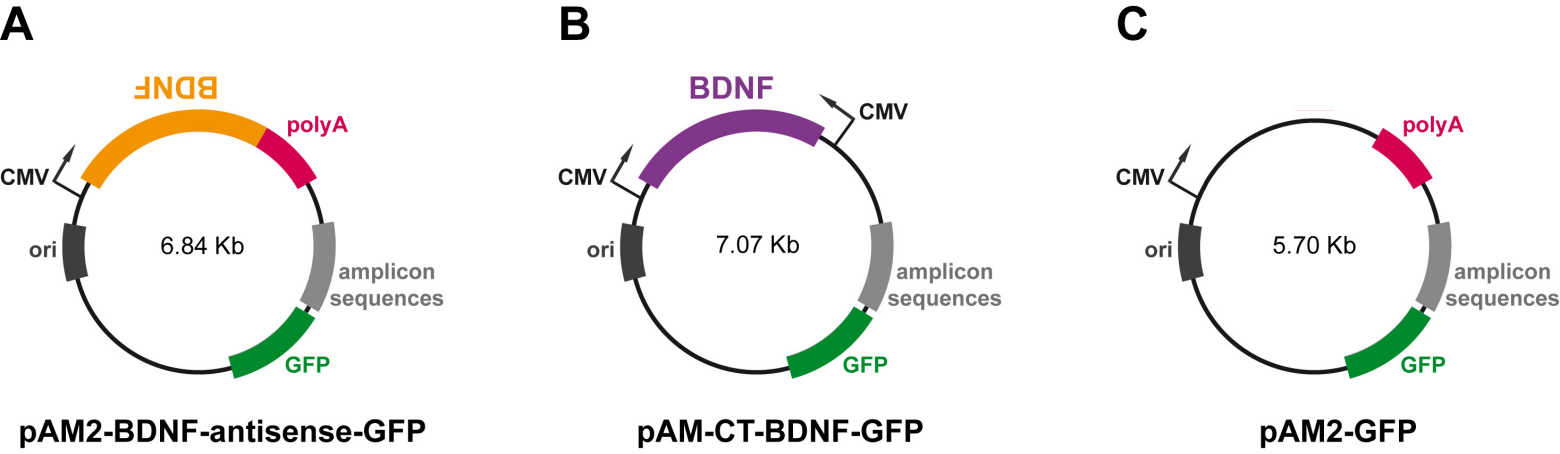
Structure of the amplicon plasmids. (A) The pAM2-BDNF-antisense-GFP plasmid (6.84 Kb) results by insertion in antisense orientation of a fragment (1.1 Kb) containing the BDNF sequence and a poly-A tail. (B) In the pAM-CT-BDNF-GFP plasmid (7.07 Kb), the BDNF sequence (1.1 Kb) is inserted in convergent transcription, between two CMV promoters. (C) The control plasmid, pAM2-GFP plasmid (5.70 Kb). These 3 plasmids were used to produce stocks of amplicon vectors at high purities (see text).

For construction of the plasmid containing antisense BDNF (plasmid pAM2-BDNF-antisense-GFP), the XbaI-BamHI fragment containing the cytomegalovirus (CMV) promoter was cut from the pMA-RQ-CMV plasmid and cloned in the XbaI-BamHI sites of pAM-GFP, a plasmid expressing the green fluorescent protein (GFP) under control of the IE4/5 promoter, to obtain the pAM-GFP-CMV plasmid. The BDNF fragment was cut from the plasmid pBSK-BDNF using EcoRI blunted-end sites by Klenow and cloned in the polylinker NheI blunted-end sites of pAM2-GFP-CMV, flanked by the CMV promoter and a Simian Virus 40 (SV40) polyadenylation signal (Fig 1A). To discriminate between cloning in sense and antisense orientation, 12 starter cultures, obtained after transformation of *E*. *Coli* high efficiency transformation competent bacteria, were digested with ScaI, PvuII and PstI, and run on agarose gel electrophoresis.

For construction of the BDNF convergent transcription plasmid (pAM-CT-BDNF-GFP), a new CMV fragment (HindIII/PmeI) was subcloned into the HindIII/EcoRV site of pAM-GFP-CMV, in the opposite direction compared to the other CMV promoter, obtaining the pAM-CT-GFP plasmid. The pAM-CT-BDNF-GFP plasmid was then obtained by cloning the EcoRI blunted-end sites by Klenow BDNF fragment of pBSK-BDNF plasmid in the EcoRV-digested pAM-CT-GFP plasmid, in order to put the BDNF sequence between the two CMV promoters (Fig 1B). The pAM2-GFP plasmid was used as control amplicon (Fig 1C).

#### Cell lines and virus

The cell lines employed in this study were the following: genetically modified mesoangioblasts producing BDNF and GFP (MABs-BDNF; [17]), Gli36 cells (a human glioblastoma cell line), Vero cells (African green monkey kidney epithelial cell line), trans-complementing Vero cells for amplification and purification of HSV-1 based amplicon vectors. All cell lines were propagated in Dulbecco’s minimum essential medium (DMEM, Lonza, Switzerland) supplemented with 10% fetal bovine serum (FBS, Invitrogen Gibco, USA), 100 U/ml penicillin and 100 mg/ml streptomycin (Invitrogen). Cells were maintained at 37°C in a humidified incubator containing 5% CO_2_. All cell lines were provided by Bioviron (France).

#### Amplicon production

Amplicon vectors were produced by transfecting 10 μg of each amplicon plasmid (pAM2-BDNF-antisense-GFP, pAM-CT-BDNF-GFP and pAM2-GFP) into trans-complementing-producing-Vero cells using the jetPRIME reagent (Polyplus-transfection, France). Cells were superinfected the following day with the LaLΔJ helper virus at a multiplicity of infection (MOI) of 0.5 plaque forming units (pfu)/cell in medium M199 (Gibco) supplemented with 1% FBS and 1% penicillin/streptomycin. Three days later, cells were harvested and amplicon viral particles were extracted by several rounds of freeze/thaw and sonication. To calculate purity of the production, amplicon and helper particles were titrated to obtain transduction units (tu)/ml (using cell number counting assay on Gli36 cells) and pfu/ml (on trans-complementing Vero cells). Several successive rounds of infections and productions were performed to obtain high quantity of amplicon particles and a final infection-production step was performed on a trans-complementing-purifying-Vero cell to obtain a final high purity working stock of amplicon vectors over the helper. The degree of purity was greater than 99% for all amplicon vectors. All virus stocks were checked for no revertant helper viruses on Vero cells.

#### Cell infection

Confluent MABs-BDNF cells seeded in 6-well plates were infected with the GFP-control, BDNF-antisense or BDNF-CT amplicon at MOI 5, and maintained at 34°C in DMEM with 10% FBS for 24, 48, 72 or 96 h. At each time point, cells were washed twice in PBS, then scraped and resuspended in 50 μl of lysis buffer (50 mM Tris-HCL pH 8, 150 mM NaCl, 1% NP-40) containing a protease inhibitor cocktail (Roche, Germany). Lysate was used for western blot analysis. The protein content of the lysates was evaluated by the Bradford method using the Bio-Rad protein assay kit (Bio-Rad Laboratories, CA, USA).

### Animals

Male Sprague-Dawley rats (240–260 g; Harlan, Italy) were used for *in vivo* experiments. They were housed under standard conditions: constant temperature (22–24°C) and humidity (55–65%), 12 h light/dark cycle, free access to food and water. Experiments involving animals were conducted in accordance with European Community (EU Directive 2010/63/EU), national and local laws and policies. The IACUC of the University of Ferrara approved this research, that was authorized by the Italian Ministry for Heath (D.M. 246/2012-B). All efforts were made to minimize animal suffering.

#### Amplicon infusion

Under ketamine and xylazine (43 and 7 mg/kg, intra-peritoneal, i.p.) anesthesia, a glass needle connected to a perfusion pump was implanted in the right dorsal hippocampus using a stereotaxic apparatus for small animals, with the following coordinates: A −1.7; L −1.5; D +3.7 [18]. Anesthesia was then maintained using isofluorane (1.4% in air, 1.2 ml/min). Two different doses of amplicon vector, 1x10^4^ tu and 5x10^5^ tu, were injected in a volume of 1 μl at a flow rate of 0.1 μl/min. Amplicon vectors (GFP-control, BDNF-antisense and BDNF-CT) were injected 5 days before pilocarpine administration.

#### Status Epilepticus

Pilocarpine was administered i.p. (340 mg/kg), 30 min after a single subcutaneous injection of methyl-scopolamine (1 mg/kg, to prevent peripheral effects of pilocarpine), and the rats’ behavior was monitored for several hours thereafter, using the scale of Racine [19]: 1, chewing or mouth and facial movements; 2, head nodding; 3, forelimb clonus; 4, generalized seizures with rearing; 5, generalized seizures with rearing and falling. Within the first hour after injection, all animals developed seizures evolving into recurrent generalized (stage 4 and higher) convulsions (status epilepticus, SE). SE was interrupted 3 h after onset by administration of diazepam (10 mg/kg i.p.). Even if it is known that long durations of pilocarpine SE lead to increased damage [20] and may even lead to lower degrees of BDNF induction [21], we chose this experimental conditions because, in our previous studies, they were associated with strong increases in BDNF levels in the hippocampus [22,23], with peak of mRNA levels at 3 h and peak of BDNF protein at 6 h after SE. Rats were therefore killed by decapitation under isofluorane anesthesia at three different time points: 3 h, 6 h and 24 h after onset of SE. Brains were rapidly frozen in 2-methylbutane.

### Histology

Brains were removed, immersed in 10% formalin for 48 h and then paraffin embedded. Serial sections of 6 μm were cut with a Microtome (Leica RM2125RT, Germany). In all experiments, adjacent sections were used for different staining procedures.

#### Immunohistochemistry

Sections were dewaxed (2 washes in xylol, 10 min each; 5 min in 100% ethanol, 5 min in 95% ethanol, 5 min in 80% ethanol) and re-hydrated in distilled water for 5 min. All antigens were unmasked using a commercially available kit (Unmasker, Diapath), according to the manufacturer’s instructions. After washing in phosphate buffered saline (PBS), sections were incubated with Triton x-100 (Sigma; 0.3% in PBS 1×, room temperature, 10 min), washed twice in PBS 1×, and incubated with 5% bovine serum albumin (BSA) and 5% serum of the species in which the secondary antibody was produced, for 30 min. They were incubated overnight at 4°C in humid atmosphere with a primary antibody specific for different cellular markers: glial fibrillary acid protein (GFAP; mouse polyclonal, Sigma) 1:100; ionized calcium binding adaptor molecule 1 (IBA-1; rabbit monoclonal, AbCam MA, USA) 1:200; GFP (rabbit polyclonal, Santa Cruz, Texas) 1:50. After 5-min rinses in PBS, sections were incubated with Triton (as above, 30 min), washed in PBS and incubated with a goat anti-mouse Alexa 594 secondary antibody (1:250, Invitrogen) for mouse primary antibodies, or with a goat anti-rabbit, Alexa 488 secondary antibody (1:250; Invitrogen) for rabbit primary antibodies, at room temperature for 3.5 hours. NeuroTrace (1:150) was included in the secondary antibody incubation. After staining, sections were washed in PBS, counterstained with 0.0001% 4’-6-diamidino-2-phenylindole (DAPI) for 15 min, and washed again. Coverslips were mounted using anti fading, water based Gel/Mount (Sigma).

For interferon-beta (IΦN–β)immunohistochemistry, we employed the Dako Cytomation EnVision^®^ + Dual Link System-HRP (DAB+) kit. Adjacent sections were unmasked as described above and, after washing in PBS, were incubated for 10 min at room temperature with Endogenous Enzyme Block to quench endogenous peroxidase activity. Subsequently, they were incubated overnight at 4°C in humid atmosphere with the primary antibody (rabbit polyclonal anti-IFN-β,1:50 dilution, MyBioSource, CA, USA). After 5-min rinses in PBS, sections were incubated for 30 min with Labeled Polymer-HRP [Dako Cytomation EnVision® + Dual Link System-HRP (DAB+)]. Staining was completed by a 5 min incubation with 3,3’-diaminobenzidine (DAB) substrated-chromogen, resulting in a brown staining of the antigen-antibody complex. Finally, coverslips were mounted using a water-based mounting medium (Gel Mount™, Sigma).

### Quantitative analysis

#### Tissue sample extraction

The left and right dorsal hippocampi were dissected by cutting the brain coronally using a metallic matrix (Zivic Instruments, PA, USA) up to a level corresponding to plate 38–57 of the rat brain atlas [18] and processed to extract total RNA, genomic DNA and proteins using the RNeasy Lipid Tissue Mini kit (Qiagen, Germany). RNA extraction was performed following the manufacturer instructions. Proteins and genomic DNA were isolated after RNA extraction using the phenol phase. Briefly, genomic DNA was precipitated from the phenol phase with ethanol and pellets were washed with sodium citrate ethanol solution and stored in 75% ethanol at -80°C. After DNA precipitation, proteins were isolated from the supernatant ethanol-phenol by isopropanol precipitation. Proteins were then washed several times with 0.3 M guanidine HCl-95% ethanol solution before being air-dried and resuspended in a rehydration buffer (62 mM Tris-HCl pH 6.8; 2% SDS; 10% glycerol; 12.5 mM EDTA; 50 mM DTT; β-mercaptoethanol; protease inhibitor cocktail) by a 20 min incubation at 95°C and 3 rounds of 30 sec sonication. The protein content of the lysates was evaluated by the Bradford method using the Bio-Rad protein assay kit (Bio-Rad Laboratories).

#### Western Blot analysis and quantification

Infected MABs and dissected dorsal hippocampal extracts, corresponding to 20 and 30 μg total proteins respectively, were analyzed by Western blotting. Proteins were quantified using the Bradford method using the Bio-Rad protein assay kit (Bio-Rad Laboratories, CA, USA) and a Bio-spectrometer (Eppendorf, Germany). Each sample was diluted in sodium dodecyl sulfate (SDS)-gel loading buffer, boiled for 10 min and centrifuged before loading. Samples were then electrophoretically separated onto a 12% SDS-polyacrylamide gel and transferred to nitrocellulose membranes. After blocking in a buffer (PBS-Tween20) containing 5% dried milk, membranes were incubated with the primary antibody in a buffer containing 2.5% dried milk overnight at 4°C. After three washings, incubations were performed with the secondary antibody in buffer/dried milk at room temperature for 1 h. The pro-BDNF protein was revealed using a rabbit anti-proBDNF monoclonal antibody (AbCam, dilution 1:1000) that is specific for pro-BDNF and does not detect mature BDNF; GFP using a mouse anti-GFP monoclonal antibody (Roche; 1:1000); actin using a rabbit anti-actin monoclonal antibody (Sigma, MO, USA; 1:1000). Mouse monoclonal antibodies were revealed using a goat anti-mouse horseradish peroxidase (HRP)-conjugated secondary antibody (Dako, Denmark; dilution 1:1000) and rabbit monoclonal antibodies by a swine anti-rabbit HRP-conjugated secondary antibody (Dako; dilution 1:3000). The immunocomplexes were detected using the ECL Western blot detection kit (GE Healthcare, NJ, USA) and ChemiDoc™ XRS (Bio-rad) for electronic blot pictures. Quantification was performed using the Image Lab software (Bio-rad).

#### Real-time quantitative polymerase chain reaction (qRT-PCR)

**Fig 2:**
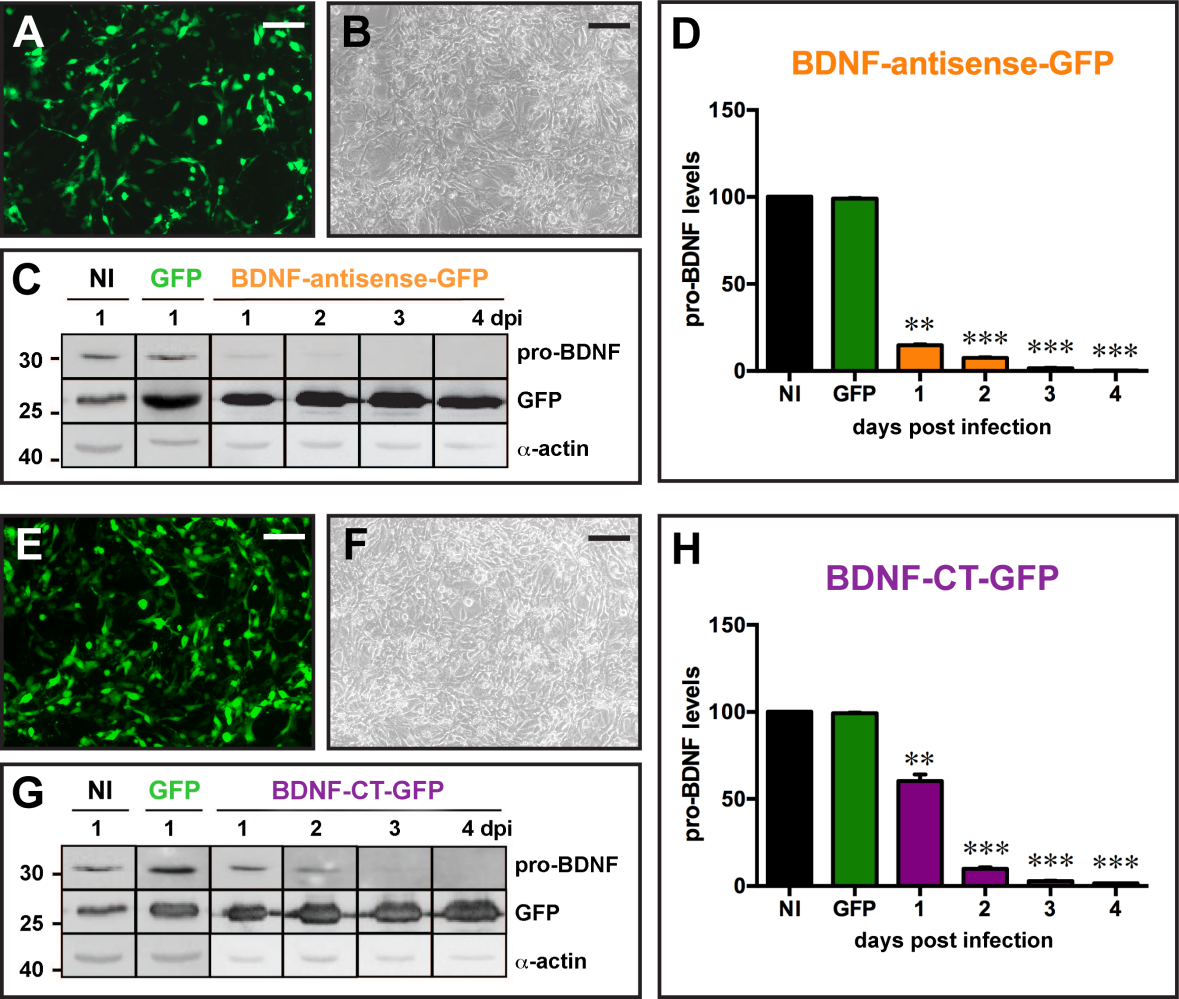
*In vitro* validation of the amplicon vectors. (A to D) Infection of mesoangioblast cells (MABs) constitutively expressing BDNF with the BDNF-antisense-GFP amplicon vector at MOI 5. Infection of the cells with amplicon vectors was confirmed by GFP fluorescence (A) and GFP detection on western blot (C). Pro-BDNF expression was analyzed by western blot in the 4 days following infection and pro-BDNF signal was normalized to α-actin for quantification (D). (E to H) Infection of MABs with the BDNF-CT-GFP amplicon vector at MOI 5. Infection of the cells was confirmed by GFP fluorescence (E) and GFP detection on western blot (G). Pro-BDNF expression was analyzed by western blot in the 4 days following infection and pro-BDNF signal was normalized to α-actin for quantification (H). Data in D and H are the mean±SEM of 6 experiments. * p<0.05, **p<0.01, ***p<0.001: ANOVA and post-hoc Dunnett test. Horizontal bars in panels A, B, E and F = 25 μm.

RNA concentration was determined using a Bio-spectrometer (Eppendorf, Germany). Strand-specific cDNA was synthetized using the cDNA first Strand Superscipt III kit (Invitrogen, USA) according to the manufacturer’s instructions, with minor modifications. Following incubation at 65°C for 5 min in ice, 500 ng total RNA from the tissue samples were reverse-transcribed with specific BDNF-antisense (AS-RT) and GAPDH primers (GAPDH-RT) at a final concentration of 0.1 μM, using the SuperScript III reverse transcriptase, at 55°C for 50 min. The AS-RT primer was designed to be specific to the amplicon sequence of the BDNF antisense mRNA and not to the endogenous BDNF or to the natural antisense BDNF sequence (Fig 2). The reaction was terminated by inactivation of the SuperScript III reverse transcriptase at 85°C for 5 min. For removing the excess of RT primers, 10 U of exonuclease I was added and the solution was incubated at 37°C for 30 min, then at 80°C for 30 min for inactivation. Next, RNase H was added and finally, following manufacturer’s instructions, each reaction was diluted to a final volume of 200 μL and cDNA stored at −20°C until use. Several controls were used, including reactions lacking RNA, reactions with RNA but lacking the reverse transcriptase enzyme and reactions lacking primers, to assess self-priming from secondary structure or genomic DNA contamination. The primers we used are listed in Table 1 and were designed using the Multipex2 Software (http://bioinfo.ut.ee/multiplx/).

**Table 1:** Primers and sequences.

	Primers	Sequences
**Reverse Transcription**	AS-RT	AATAGCATCACAAATTTCACAA
	GAPDH-RT	TGGTCCAGGGTTTCTTACTC
**qPCR**	qPCR AS For	AATTCACGCGTGGTACCTCTA
	qPCR AS Rev	CACTGCATTCTAGTTGTGGTTTG
	qPCR ratGAPDH For	GGGTGTGAACCACGAGAAAT
	qPCR ratGAPDH Rev	ACTGTGGTCATGAGCCCTTC

BDNF-antisense was assessed by qRT-PCR on a Bio-Rad CFX96 Real-Time PCR Detection System (Bio-rad) using SSoAdvanced Universal SYBR Green Supermix (Bio-Rad Laboratories). Primers were designed using the Prirmer3 Plus Software (http://www.bioinformatics.nl/cgi-bin/primer3plus/primer3plus.cgi) according to Bio-Rad instructions. Their sequences are described in Table 1. The BDNF antisense qPCR primers amplify specifically the amplicon sequence of the antisense BDNF mRNA. Two μL of the first-strand cDNA sample or reverse transcription controls described above were used in the reaction, and water was used for negative control reaction. Each qPCR experiment was performed in triplicate for both controls and test samples. The qPCR reactions contained the SsoAdvanced universal SYBR Green supermix, plus 250 nM or 500 nM of forward and reverse primers for GAPDH or BDNF-antisense, respectively. The qPCR conditions for all targets were as follows: 95°C for 30 s, then 95°C for 10 s and 64°C for 20 s repeated 40 times. At the end of the run, the Melting curve analysis was 60°C to 95°C with 0.5°C increment and 2–5 s per step. Data analyses were done with CFX Manager (Bio-Rad) and real-time data quantification was achieved using the ∆Ct values using GAPDH as reference gene. Each RNA sample was run in a separate assay: 14 samples of dorsal hippocampi injected with the BDNF-antisense-GFP amplicon vector, 15 injected with the control vector and 3 not treated at all (naïve).

### Statistical analysis

Statistical comparisons of the data were performed using ANOVA and *post-hoc* the Dunnett’s test for *in vitro* validation of amplicon vectors, to compare pro-BDNF expression in the right and left hippocampus, and to determine whether the time to enter convulsive SE differed significantly between rats injected with the different amplicon vectors.

## Results

### HSV-1 amplicon plasmid generation

We developed two silencing strategies to down-regulate BDNF protein level. The antisense strategy targets and degrades the cytoplasmic mRNA pool of BDNF. For this purpose, we generated an amplicon plasmid, pAM2-BDNF-antisense-GFP, that expresses both the mRNA for GFP and the synthetic antisense rat BDNF mRNA (Fig 1A). The second strategy, based on the Convergent Transcription (CT) technology, represses the *BDNF* gene at the level of transcription. For this purpose, we generated a plasmid (pAM2-CT-BDNF-GFP) in which the BDNF cDNA is inserted between two cytomegalovirus (CMV) promoters oriented in opposing directions (Fig 1B), such that the resulting convergent transcription could elicit down-regulation of the *BDNF* gene transcription through chromatin remodeling associated with epigenetic silencing marks [14]. We also generated a control amplicon plasmid and vector (named GFP amplicon vector), which possesses only the GFP reporter cassette (Fig 1C).

Following the cloning steps, large purified stocks of 3 corresponding amplicon vectors were produced. The titer of each stock was 9.4×10^8^ t.u./ml, 1.05×10^9^ t.u./ml and 1.05×10^7^ t.u./ml, respectively for BDNF-antisense-GFP, BDNF-CT-GFP and control GFP amplicon vectors. The BDNF-antisense-GFP and BDNF-CT-GFP amplicon vectors contain more than 20 copies of the silencing cassette. As described, we produced each amplicon vector with a GFP expression cassette for monitoring the infection in cells and animals.

### *In vitro* validation

The next step was to evaluate the effect of each amplicon vector against BDNF *in vitro*. In order to study efficiency to repress BDNF expression, we infected BDNF-expressing mesoangioblast (MABs) cells with BDNF-antisense-GFP or BDNF-CT-GFP amplicon vectors at a multiplicity of infection (MOI) of 5. Control conditions were MAB cells infected with the control GFP amplicon vector at MOI 5 or not infected. The infection of cells with BDNF-antisense-GFP or BDNF-CT-GFP amplicon vectors was confirmed by a strong GFP fluorescence, as shown in Fig 2A and 2E. As an outcome measure to assess effectiveness of the vectors, we examined levels of the precursor protein for BDNF, i.e. pro-BDNF, for 96 hours after infection using western blot analysis. MAB cells infected with the BDNF-antisense-GFP amplicon vector displayed a strong reduction of pro-BDNF levels beginning as soon as 24 h post infection as compared with uninfected cells or cells infected with the control GFP amplicon vector. The decrease of pro-BDNF protein levels was even more prominent at the following time-points, and became essentially complete 96 h after infection (Fig 2D). These data indicate a fast and very efficient silencing activity for the BDNF-antisense-GFP amplicon vector *in vitro*.

The same experiment was performed using the BDNF-CT-GFP amplicon vector and, again, we observed a nearly complete cancellation of pro-BDNF expression from MAB cells at 96 h after the injection (Fig 2G). However, the time-course differed in that the decline in pro-BDNF protein levels occurred more slowly than with BDNF-antisense-GFP (Fig 2H). These results indicate that, *in vitro*, both amplicon vectors, BDNF-antisense-GFP and BDNF-CT-GFP, produce a highly efficient knock-down of pro-BDNF levels. Thus, both vectors were elected for *in vivo* testing.

### *In vivo* validation

**Fig 3:**
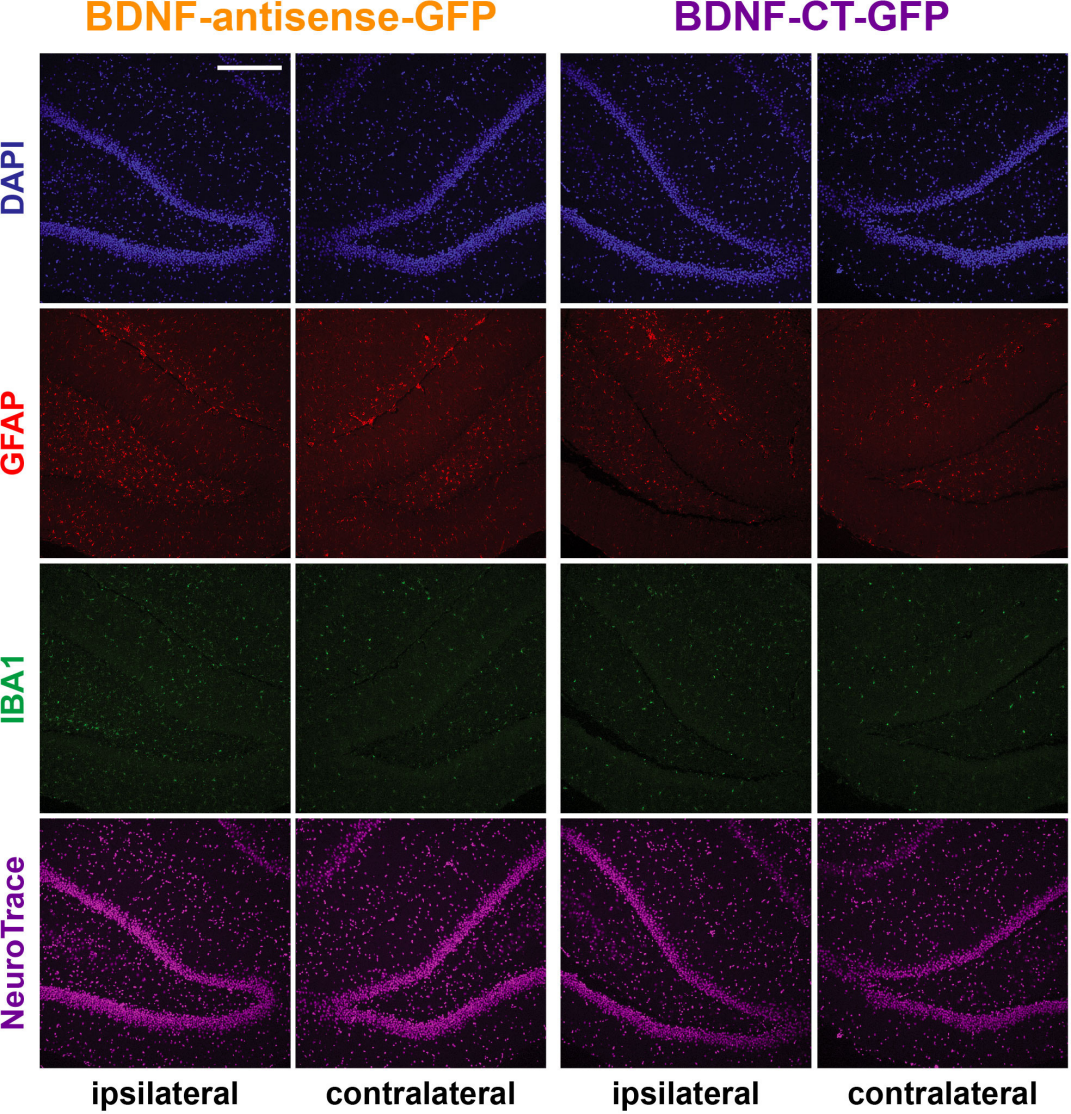
Absence of overt amplicon vector-induced toxicity after injection in the dorsal hippocampus. Dentate gyrus (DG) of the dorsal hippocampus injected (ipsilateral) and non-injected (controlateral) with BDNF-antisense-GFP or with BDNF-CT-GFP amplicon vector. Nuclei are marked by DAPI in blue, GFAP-positive astrocytes in red, IBA-1-positive microglia in green and neuronal nuclei are labeled by NeuroTrace in magenta. Horizontal bars = 100 μm.

We first explored the toxicity of the amplicon vectors after direct injection in the rat hippocampus. To this aim, we injected 5×10^5^ t.u. of either vector in a volume of 1 μl in the right hippocampus dentate gyrus area of naïve rats and, 5 days after injection, examined gliosis, microcytosis and neuronal loss using GFAP, IBA-1 immunofluorescence and NeuroTrace staining, respectively. Administration of the BDNF-antisense-GFP or of the BDNF-CT-GFP amplicon vectors did not alter the morphology of the hippocampus (Fig 3 shows the dentate gyrus; S1 Fig shows the whole hippocampus). The density of GFAP-positive cells in the injected hippocampus was similar to that of the non-injected, contralateral one, i.e. there was no indication of reactive astrocytosis (Fig 3 and S1 Fig). Similar to GFAP cells, the density of IBA-1 positive cells was comparable in both the ipsilateral and the contralateral hippocampus, indicating absence of reactive microgliosis (Fig 3 and S1 Fig). Finally, neuronal density, as measured using the NeuroTrace staining, was also not altered after injection of either amplicon vector (Fig 3 and S1 Fig). Taken together, these data suggest that treatment with amplicon vectors do not induce an overt damage when directly injected in the brain.

We also evaluated the interferon response by using IFN-β immunohistochemistry. IFN-β positive cells were detected at the site of injection of BDNF-antisense-GFP, and the majority of these cells appeared to be microglial based on IBA-1 immunofluorescence (S2A Fig). This finding is in line with previous literature reports [24,25]. IFN-β positive cells were also detected after injection of the BDNF-CT-GFP amplicon vector and, interestingly, the IFN response seemed greater than with BDNF-antisense-GFP (S2B Fig). This finding may be due to higher levels of double stranded RNA generated with the CT technology, as compared with the antisense approach.

**Fig 4:**
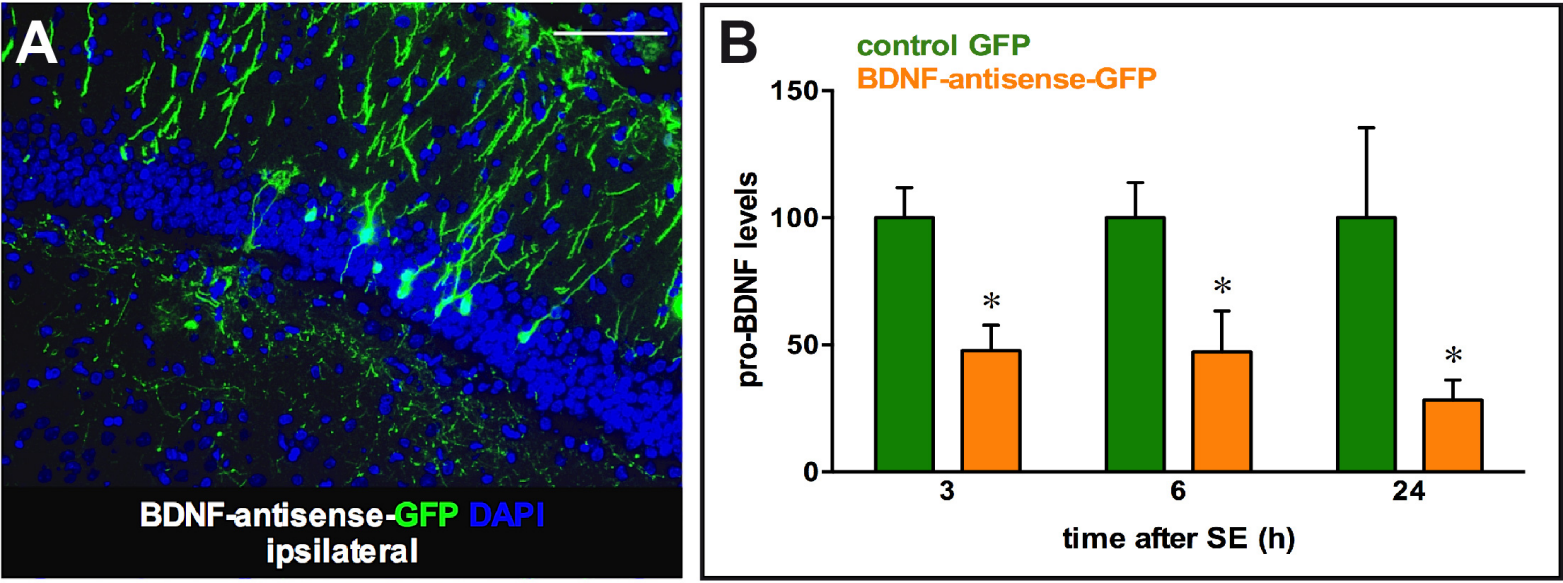
Transgene expression following injection of amplicon vectors in the right and left hippocampus at different time points after pilocarpine-induced status epilepticus. (A) Representative GFP immunofluorescence in the dorsal hippocampus of a rat at 5 days post injection with the BDNF-antisense-GFP amplicon vector. (B) Quantification of the pro-BDNF signal, normalized to α-actin, 3, 6 and 24 h after pilocarpine status epilepticus induced 5 days after injection of the amplicon vectors in the right dorsal hippocampus. Data in B are the mean±SEM of 4–5 rats per group. * p<0.05, ANOVA and post-hoc Dunnett test. Horizontal bar in A = 250 μm.

Next, we tested the biological efficiency for down-regulation of BDNF protein levels. To this aim, we decided to employ the pilocarpine model. Intra-peritoneal injection of pilocarpine in rodents provokes generalized seizures leading to a status epilepticus (SE), which drives a massive increase in BDNF levels in the hippocampus [26,22]. To test the efficiency of amplicon vectors, we injected them in the right dorsal hippocampus, 5 days before pilocarpine administration, and animals were then killed at 3 different time points: 3 h (peak of pilocarpine-induced increase in BDNF mRNA levels; [27,26]), 6 h (peak of pilocarpine-induced increase in BDNF protein levels; [28,22]) and 24 h after onset of SE. Rats were injected either with the control GFP, the BDNF-antisense-GFP or the BDNF-CT-GFP vector at 2 doses, 1×10^4^ or 5×10^5^ t.u., i.e. the high dose that did not cause overt toxicity (see above) and a >1 log lower dose. Amplicon vector injections into the dorsal hippocampus produced expression of GFP in infected cells (Fig 4A for BDNF-antisense-GFP) at the site of injection, whereas a negligible number of positive cells were observed contralateral to injection under these experimental conditions. We therefore decided to use the contralateral dorsal hippocampus as an internal control. First, we quantified synthetic antisense BDNF mRNA levels in the ipsilateral and contralateral hippocampi injected with the BDNF-antisense-GFP amplicon vector, using the real-time quantitative polymerase chain reaction (qRT-PCR) method and primers specific to the amplicon cassette for the synthetic BDNF antisense mRNA but absent in the endogenous sense and natural antisense BDNF mRNA (S3 Fig). Amplicon-derived synthetic antisense BDNF mRNA was detected in dorsal hippocampi injected with the BDNF-antisense-GFP amplicon vector (mean ∆CT 1.4±0.7; n = 14), but not in the contralateral, non-injected side (no detectable signal). In addition, the synthetic antisense BDNF mRNA was neither detectable in animals injected with the control vector (n = 15) nor in naïve animals (n = 3).

Pro-BDNF expression was then measured by western blot in the hippocampus at 3 different time points after SE, and the signal was normalized to α-actin before calculating the ratio between the ipsilateral (right) and contralateral (left) hippocampus. The control GFP amplicon vector did not produce any effect, whereas the low dose (1×10^4^ t.u.) of the BDNF-antisense-GFP amplicon vector exhibited a robust reduction of pro-BDNF protein levels at all time points (Fig 4B). This effect may be dose-dependent, because the high dose led to a greater (even if not significantly greater) effect. Different from the antisense strategy, only a small, non-significant reduction (about 10%) in pro-BDNF protein levels was observed in low (1×10^4^ t.u.) or high (5×10^5^ t.u.) dose BDNF-CT-GFP injected hippocampi (low dose effect at 3 h after SE shown in S4 Fig). Thus, the BDNF-CT-GFP amplicon vector cannot induce sufficiently robust and/or prolonged heterochromatin changes to prevent SE-related *BDNF* transactivation *in vivo*, whereas the BDNF-antisense amplicon vector proves effective in knocking down efficiently pro-BDNF protein levels.

**Fig 5:**
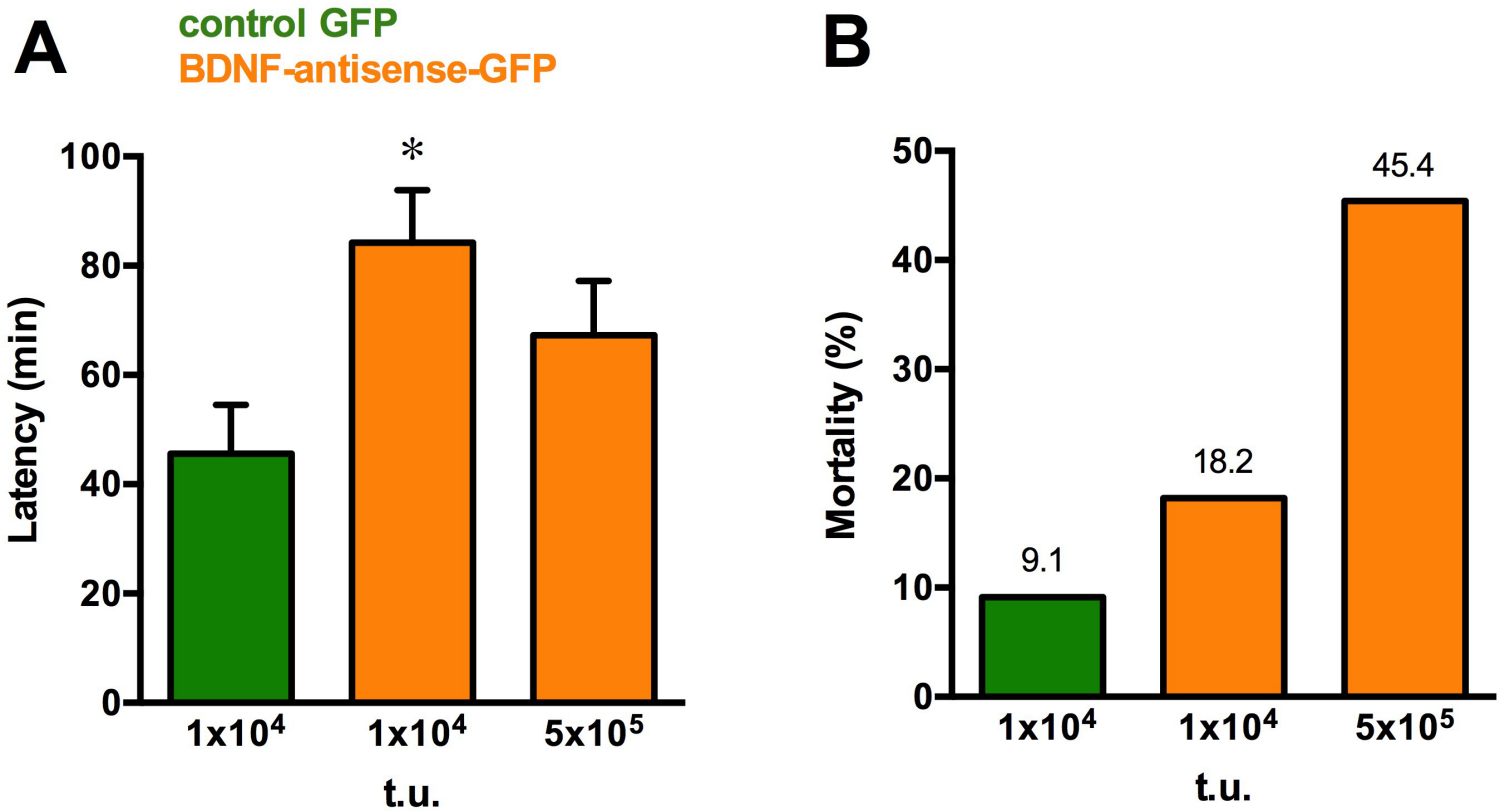
Behavioral effects. (A) Time to enter convulsive status epilepticus following administration of the different doses of BDNF-antisense-GFP vector. * p<0.05, ANOVA and post-hoc Dunnett test. (B) Mortality of pilocarpine-treated animals injected with the different doses of BDNF-antisense-GFP amplicon vector. Data in are the mean±SEM of 10–14 animals.

The model system we employed for analysis of *BDNF* knock down permits an initial evaluation of the behavioral implications. Knocking down *BDNF* overexpression only in a part of a single hippocampus cannot be expected to produce robust behavioral effects in a model in which a pro-convulsant agent is administered systemically. However, we observed that animals treated with BDNF-antisense-GFP entered convulsive SE later then those treated with the control GFP amplicon vector (Fig 5A). Moreover, whereas the infusion of both BDNF-antisense-GFP and BDNF-CT-GFP amplicon vectors per se did not produce overt signs of behavioral toxicity, because all animals were apparently well after the surgery and in the following days, the percentage of animals that died after pilocarpine administration was higher in the high dose BDNF-antisense group (Fig 5B). Thus, even knocking down *BDNF* expression in a portion of a single hippocampus seems sufficient to elicit significant behavioral effects.

## Discussion

In this study, we generated two types of amplicon vectors to locally knock down the levels of BDNF: a classical antisense approach and an approach based on convergent transcription. The latter technology was used for the first time in combination with a viral vector, both *in vitro* and *in vivo*. Whereas both approaches proved highly efficient *in vitro* with a nearly complete knock down of BDNF protein, only the former (antisense) provided robust results in the *in vivo* settings of SE-induced *BDNF* overexpression in the hippocampus.

HSV-1-based amplicon particles were generated following a recently described method that produces relatively high titers of vector stocks with reduced amounts of helper virus [29]. Although this helper virus is completely defective, cannot replicate in cells nor it can disseminate in organisms, it was important to highly purify the amplicon vectors so to prevent all potential residual cytotoxicity. Here, we produced amplicon vectors with very low helper contamination (less than 1%) and provided evidence that these vectors were not cytotoxic and produced no overt cell damage when injected *in vivo*, although subtle apoptotic cell death cannot be ruled out. Other advantages of the amplicon approach are also significant. In particular, worth of note is the fact that the amplicon genomes are free of HSV-1 genes and replication functions, offering the opportunity to package very large transgenic sequences: it is thus possible to transduce more than 110 kb of foreign DNA in a single particle [30]. Since this amount of space (152 kbp total) must all be filled to produce stable particles, small transgenes are repeated in concatameters, with a number of repeats that will depend on the size of the original amplicon plasmid [5,31]. In the present study, the produced BDNF-antisense-GFP and BDNF-CT-GFP amplicon vectors contained more than 20 copies of the BDNF silencing cassette and therefore were expected to ensure a particularly efficient knock down of the protein of interest.

HSV-1 based amplicon vectors also share many useful features of the HSV-1 parent virus [32], like the ability to infect a broad range of dividing and non-dividing host cells, including neurons. HSV-1 based amplicon vectors allow efficient infection of many neuronal types, with transgene expression over an extended period of time without demonstrable side effects [33].

The two strategies for knocking down *BDNF* gene expression, the classical antisense strategy and the convergent transcription strategy, are conceptually different. The former is based on the technique of RNA interference (RNAi) that was first discovered in *Caenorhabditis elegans* [6]. RNAi is a gene silencing mechanism in which a double-stranded RNA (dsRNA) molecule is generated that directs the specific degradation of the corresponding target mRNA [34]. The BDNF-antisense-GFP amplicon targets and degrades the cytoplasmic mRNA pool of BDNF through the mechanism of RNA interference: expression of the transgene leads to formation of sense RNA-RNA hybrids in the cytosol and thereby degrades and/or prevents translation of pro-BDNF mRNA. The second strategy that we tested is based on the convergent transcription (CT) technology [9] that acts through nuclear transcriptional gene silencing. The CT technology seemed particularly attractive since it has been reported to induce locally restricted heterochromatin spreading at the target gene and a silencing with long-lasting effects compared to the transient siRNA or shRNA methods [14]. The *in vitro* experiments demonstrated a reliable and very strong effect of both amplicon vectors in knocking down pro-BDNF levels and, therefore, both were elected for *in vivo* studies. Noteworthy, the kinetics of BDNF reduction seemed slower with the CT compared with the antisense strategy.

To study the effects of amplicon *in vivo*, we decided to employ a status epilepticus model. Epileptogenic stimuli are known to affect the expression of BDNF transcripts in the hippocampus [35,23]. Under the experimental conditions employed in this study, pilocarpine leads to increased BDNF mRNA and protein levels peaking respectively 3 and 6 h after onset of status epilepticus [22,28]. It is thought that increased BDNF levels play a role in the transformation of a normal brain in epileptic, i.e. in a brain that can spontaneously generate seizures. Indeed, spontaneous seizures begin to occur a few days after pilocarpine status epilepticus, and this latency period is associated with plastic changes in the epileptogenic area, including increased neurogenesis, cell death, plastic modifications of synaptic contacts [36]. BDNF exerts relevant effects upon all of these phenomena. However, it is still unclear what the implications of its increased biosynthesis could be. Strategies to down-regulate the BDNF signal have been reported by many to retard epileptogenesis [37–40], and bath-applied BDNF exacerbates seizure activity in the epileptic hippocampus *in vitro* [41,42]. In contrast, the combined supplementation of BDNF and fibroblast growth factor 2 (FGF-2) has been reported to attenuate cellular alterations associated with epileptogenesis and to highly significantly reduce the frequency and severity of spontaneous recurrent seizures [43–45]. To dissect out these mechanisms there is a need to develop tools to locally modulate the BDNF signal *in vivo*, in particular to block it.

Therefore, we decided to test the ability of our vectors to down-regulate BDNF expression in the pilocarpine model system. First, in keeping with previous reports [33], we found that *in vivo* injection of amplicon vectors did not cause obvious toxic effects (cell death) nor significant activation of astrocytes or microglia. Second, and more important, we found that the silencing activity mediated by the BDNF-antisense-GFP amplicon vector was highly significant, even at relatively low doses, whereas the BDNF-CT-GFP amplicon vector did not produce significant reductions in pro-BDNF levels. Why the efficiency of *in vivo* silencing was so different between the two strategies, in spite of the fact that both were equally effective *in vitro*, is difficult to understand. One hypothesis could be that CT cannot induce sufficiently strong and/or prolonged heterochromatin changes on the genome to prevent pilocarpine-induced transactivation of the *BDNF* gene. On the contrary, antisense RNAi-based mechanisms would be more efficient and rapid at degrading a pool of cytoplasmic BDNF mRNA, thus leading to a stronger and a more prolonged effect on BDNF synthesis.

Importantly, the knock-down of BDNF levels induced with BDNF-antisense-GFP was sufficient to produce significant behavioral effects, in spite of the fact that it was produced in a part of a single hippocampus and not in the entire epileptogenic area. Moreover, the kind of behavioral results that were obtained are also worth noting, in that they reflect the double-edge pattern of BDNF effects in epileptogenesis. On one hand, consistent with the pro-epileptic effects of BDNF [37–40] we observed an increased latency to onset of status epilepticus in BDNF-antisense-GFP-injected animals. On the other hand, consistent with the neuroprotective role of BDNF [45] we observed an increased mortality of animals injected with BDNF-antisense-GFP. These initial data must be verified and extended using multiple, bilateral injections ensuring a robust and widespread knock down of *BDNF* gene expression in the epileptogenic region.

In conclusion, this study demonstrates a reliable effect of amplicon vectors in knocking down gene expression. At variance with the CT strategy, which is effective only *in vitro*, the BDNF-antisense-GFP amplicon vector proves effective both *in vitro* and *in vivo*, knocking down efficiently BDNF protein levels in the injected hippocampus at different time points. Therefore, the antisense strategy seems a better choice for silencing BDNF expression *in vivo*, al least in our model. This is further supported by the observation that even knocking down BDNF expression in a portion of a single hippocampus is sufficient to elicit significant behavioral effects. Taken together, these data illustrate the broad potential of HSV-1 based amplicon vectors as gene transfer tools for effectively silencing gene expression.

## Supporting Information

S1 Fig Absence of overt amplicon vector-induced toxicity after injection in the dorsal hippocampus. Dorsal hippocampus injected (ipsilateral) and non injected (contralateral) with BDNF-antisense-GFP or with BDNF-CT-GFP amplicon vector. Nuclei are marked by DAPI in blue, GFAP-positive astrocytes in red, IBA-1-positive microglia in green and neuronal nuclei are labeled by NeuroTrace in magenta. Horizontal bars = 200 μm (12,5 μm in CA1/CA3 boxes).
(TIF)

S2 Fig Interferon β response. Representative sections showing IFN-β immunohistochemistry in the dorsal hippocampus injected with the BDNF-antisense-GFP (left panel, A) or with the BDNF-CT-GFP amplicon vector (right panel, B). In the insert, nuclei are marked in blue by DAPI and IBA-1-positive cells (microglia) are in red. Horizontal bar = 25 μm.
(TIF)

S3 Fig Structure of the qRT-PCR primers. (A) Synthetic antisense BDNF mRNA. (B) Endogenous BDNF mRNA. CDS: coding DNA sequence. UTR: untranslated region. Rev: reverse. For: forward.
(TIF)

S4 Fig Transgene expression following injection of amplicon vectors in the right and left hippocampus at 3h after pilocarpine-induced status epilepticus. (A) Representative GFP immunofluorescence in the dorsal hippocampus of a rat at 5 days post injection with the BDNF-CT-GFP amplicon vector. (B) Quantification of the pro-BDNF signal, normalized to α-actin, 3 h after pilocarpine status epilepticus induced 5 days after injection of the amplicon vectors in the right dorsal hippocampus. (n = 5 animals per group). Horizontal bar in A = 250 μm.
(TIF)
