# Supplementary figures and images for: Silencing Status Epilepticus-Induced BDNF Expression with Herpes Simplex Virus Type-1 Based Amplicon Vectors
Source: PLoS One. 2016 Mar 8;11(3):e0150995. doi: 10.1371/journal.pone.0150995 (PMC4783051; doi:10.1371/journal.pone.0150995)

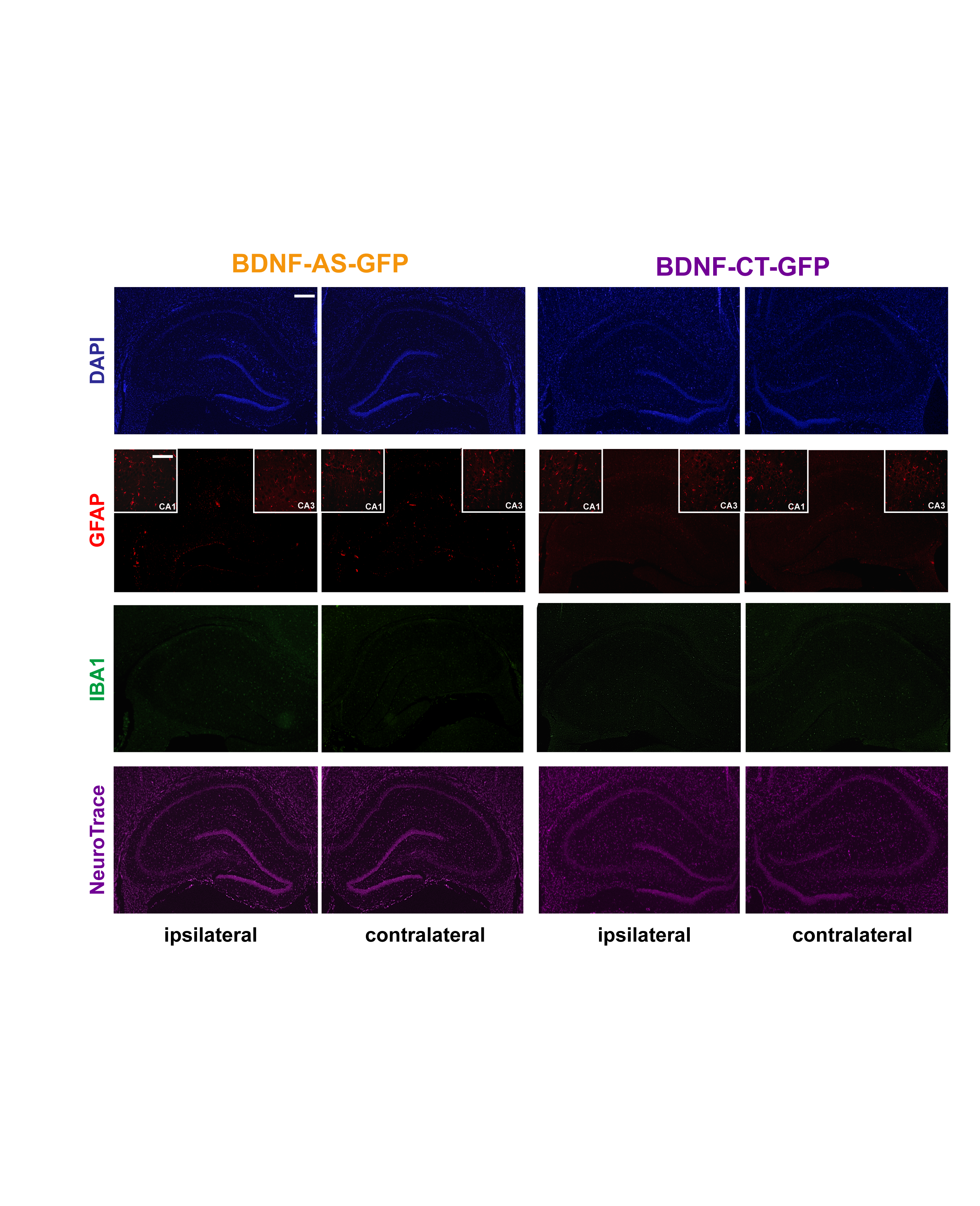

Supplement: S1 Fig — Dorsal hippocampus injected (ipsilateral) and non injected (contralateral) with BDNF-antisense-GFP or with BDNF-CT-GFP amplicon vector. Nuclei are marked by DAPI in blue, GFAP-positive astrocytes in red, IBA-1-positive microglia in green and neuronal nuclei are labeled by NeuroTrace in magenta. Horizontal bars = 200 μm (12,5 μm in CA1/CA3 boxes). (TIF) [file pone.0150995.s001.tif]

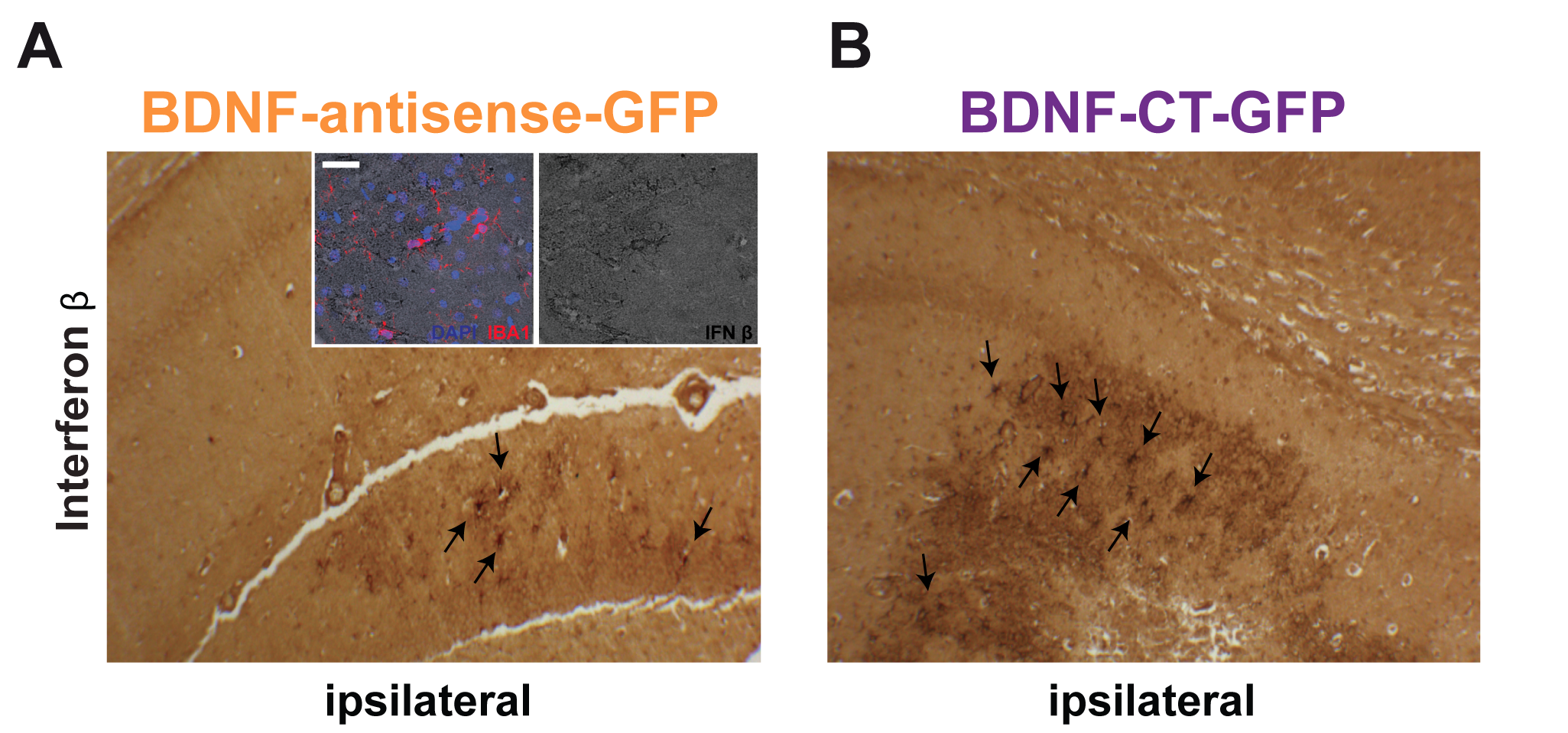

Supplement: S2 Fig — Representative sections showing IFN-β immunohistochemistry in the dorsal hippocampus injected with the BDNF-antisense-GFP (left panel, A) or with the BDNF-CT-GFP amplicon vector (right panel, B). In the insert, nuclei are marked in blue by DAPI and IBA-1-positive cells (microglia) are in red. Horizontal bar = 25 μm. (TIF) [file pone.0150995.s002.tif]

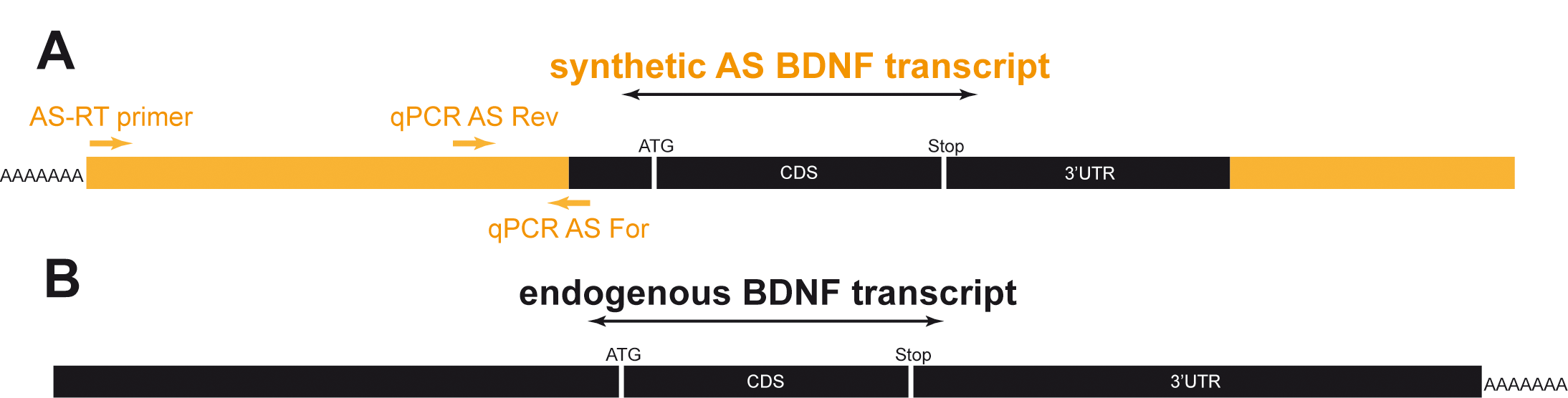

Supplement: S3 Fig — (A) Synthetic antisense BDNF mRNA. (B) Endogenous BDNF mRNA. CDS: coding DNA sequence. UTR: untranslated region. Rev: reverse. For: forward. (TIF) [file pone.0150995.s003.tif]

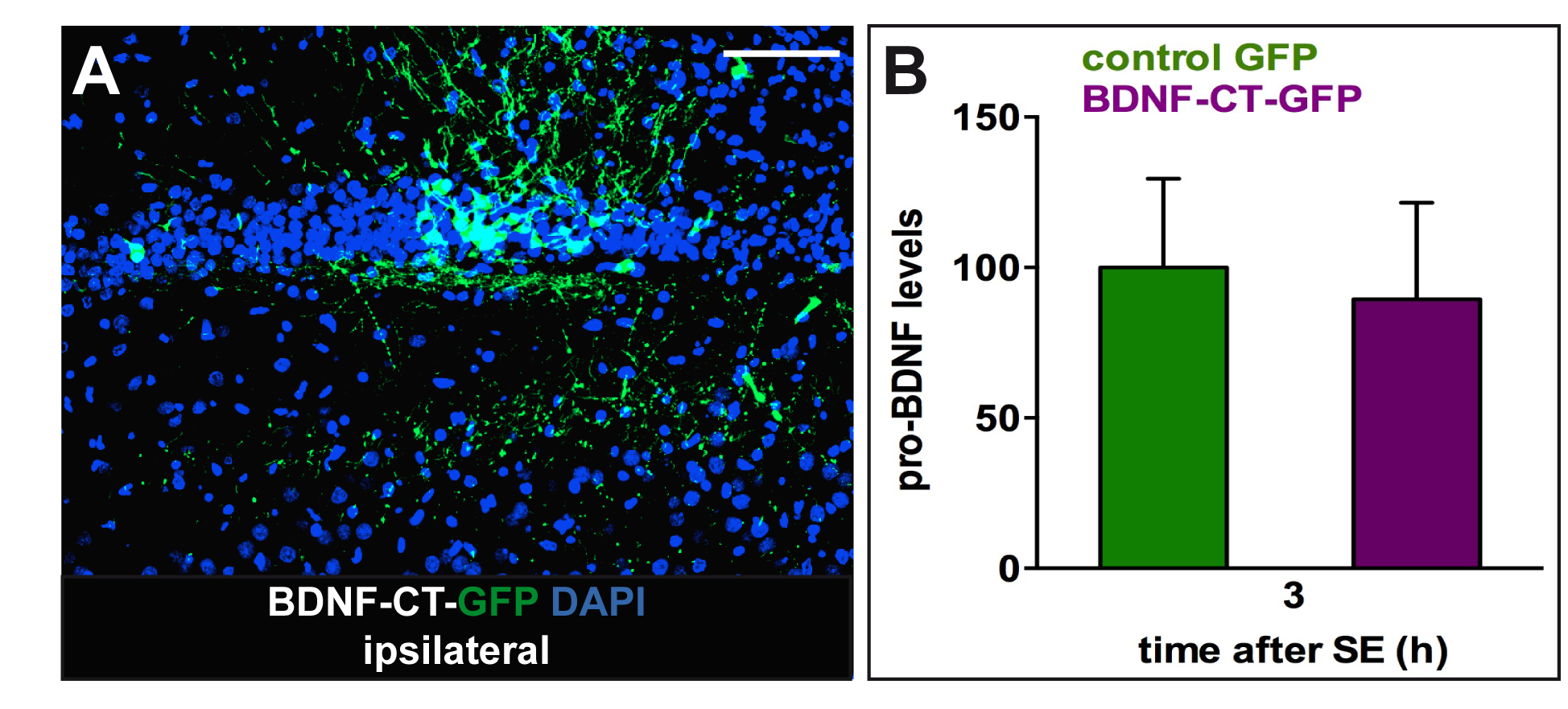

Supplement: S4 Fig — (A) Representative GFP immunofluorescence in the dorsal hippocampus of a rat at 5 days post injection with the BDNF-CT-GFP amplicon vector. (B) Quantification of the pro-BDNF signal, normalized to α-actin, 3 h after pilocarpine status epilepticus induced 5 days after injection of the amplicon vectors in the right dorsal hippocampus. (n = 5 animals per group). Horizontal bar in A = 250 μm. (TIF) [file pone.0150995.s004.tif]
